# Transmural perfusion gradient analysis by high-resolution MR versus fractional flow reserve for the assessment of coronary artery stenosis

**DOI:** 10.1186/1532-429X-14-S1-O90

**Published:** 2012-02-01

**Authors:** Amedeo Chiribiri, Gilion Hautvast, Tim Lockie, Andreas Schuster, Boris Bigalke, Luca Olivotti, Simon Redwood, Marcel Breeuwer, Sven Plein, Eike Nagel

**Affiliations:** 1 Imaging Sciences and Biomedical Engineering, King's College London, London, UK; 2 Imaging Systems - MR, Philips Healthcare, Best, Netherlands; 3 Cardiovascular Division, King's College London, London, UK; 4 Biomedical Image Analysis, University of Technology, Biomedical Engineering, Eindhoven, Eindhoven, Netherlands; 5 Division of Cardiovascular and Neuronal Remodeling, University of Leeds, Leeds, UK

## Summary

We present the results of transmural perfusion gradient (TPG) analysis (a novel method to evaluate the presence of subendocardial ischemia) versus fractional flow reserve (FFR) for the detection of hemodynamically significant coronary artery disease (CAD).

## Background

The subendocardial layer of the left ventricle is affected earlier and more severely by ischemia as a consequence of the interaction between coronary microvasculature and cardiac contraction. The identification of subendocardial ischemia is thus considered a sensitive and specific endpoint for the diagnosis of CAD. TPG due to subendocardial ischemia can be visualised on high resolution perfusion cardiovascular magnetic resonance (CMR) images and their presence can be specifically assessed by the gradientogram plot. This study tests the hypothesis that transmural perfusion gradients by adenosine stress CMR predict hemodynamically significant CAD as assessed by FFR and compares TPG with perfusion quantitative analysis.

## Methods

63 patients (49 male, 60±9 years) with known or suspected CAD underwent high-resolution (1.2 x 1.2 mm in plane) adenosine stress perfusion CMR at 3.0T. FFR was measured in all vessels with >50% severity stenosis. FFR<0.80 was considered hemodynamically significant. TPG were measured by the gradientogram plot and data analysed based on different thresholds of transmural perfusion redistribution (of 5%, 10%, 15% and 20%). Myocardial perfusion reserve (MPR) was assessed by Fermi deconvolution.

## Results

In the first group of 28 patients, who served as a training group, a 15% TPG was identified as the best diagnostic threshold for FFR<0.80. Lower (5% and 10%) and higher thresholds (20%) resulted in less accurate results (Table 1). A 15% threshold was then tested prospectively in the second group of 35 patients and showed a high accuracy in the detection of hemodynamically significant stenoses (n=105 vessels; area under the curve 0.893; sensitivity 0.88, specificity 0.91). Intra- and interobserver variability were good (κ=0.84 and κ=0.72, respectively).

**Table 1:** Transmural perfusion analysis details in Group 1 for thresholds of 5%, 10%, 15% and 20%.

	5% threshold	10% threshold	15% threshold	20% threshold
Sensitivity	0.88 (0.60-0.98)	0.81 (0.54-0.95)	0.81 (0.57-0.93)	0.63 (0.36-0.84)

Specificity	0.56 (0.44-0.68)	0.75 (0.63-0.84)	0.85 (0.74-0.91)	0.93 (0.84-0.97)

Positive Predictive Value	0.31 (0.19-0.47)	0.42 (0.25-0.61)	0.54 (0.33-0.74)	0.67 (0.39-0.87)

Negative Predictive Value	0.95 (0.83-0.99)	0.95 (0.84-0.99)	0.95 (0.86-0.99)	0.92 (0.92-0.96)

Area under the ROC curve	0.72 (0.59-0.85)	0.78 (0.65-0.91)	0.83 (0.71-0.95)	0.78 (0.63-0.93)

On ROC analysis performed on Group 1, a MPR cut-off of 1.55 provided optimal diagnostic accuracy to detect myocardial ischemia (area under the ROC curve 0.81). When the same MPR cut-off was applied to the quantitative analysis of Group 2, it resulted in a sensitivity of 0.70 and a specificity of 0.91. On ROC analysis, the AUC was 0.82. Gradient analysis was more accurate than myocardial perfusion reserve (p=0.001).

## Conclusions

The detection of transmural perfusion gradients by high-resolution CMR allows an accurate diagnosis of hemodynamically significant CAD as compared to FFR and in this study was more accurate than myocardial perfusion reserve.

## Funding

The Centre of Excellence in Medical Engineering funded by the Wellcome Trust and the Engineering and Physical Sciences Research Council (EPSRC) under grant number WT 088641/Z/09/Z. Andreas Schuster received grant support from the British Heart Foundation (BHF) (RE/08/003 and FS/10/029/28253) and the Biomedical Research Centre (BRC-CTF 196). Sven Plein was funded by a Wellcome Trust Research Fellowship during the early phase of this study (WT078288).and is currently funded by a British Heart Foundation Senior Research fellowship (FS/10/62/28409). Timothy Lockie was funded by a British Heart Foundation Clinical Research Training Fellowship (FS/08/058/25305).

**Figure 1:**
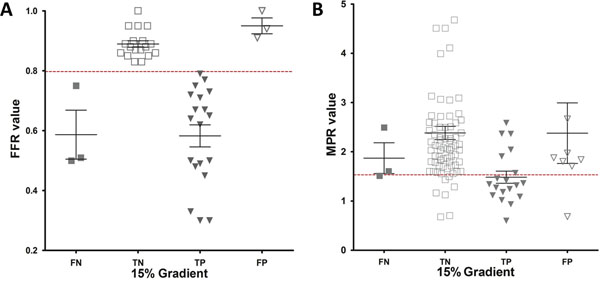
(A) Scatterplot showing the distribution of FFR values according to the results of transmural perfusion gradient analysis (FN: false negative; TN: true negative; TP: true positive; FP: false positive). (B) Scatterplot showing the distribution of MPR values according to the results of transmural perfusion gradient. A dichotomous cut-off of 0.80 was used to signify a significant lesion.

